# Selenomethionine Alleviates Zearalenone-Induced Liver Injury in Rabbits Through SIRT1-FOXO1/P53 Signaling Pathway

**DOI:** 10.3390/antiox15020176

**Published:** 2026-01-30

**Authors:** Xiaoguang Chen, Wenjuan Wei, Haonan Li, Wenjing Xu, Qiongxia Lv, Yumei Liu, Ziqiang Zhang

**Affiliations:** College of Animal Science and Technology, Henan University of Science and Technology, Luoyang 471023, China; wwj3456453421@163.cm (W.W.); lihaonan09@163.com (H.L.); xwenjing0223@163.com (W.X.); lvqx20001@163.com (Q.L.); yumeiliiu@haust.edu.cn (Y.L.); ziqiangzhang@haust.edu.cn (Z.Z.)

**Keywords:** zearalenone, selenomethionine, liver injury, oxidative stress, apoptosis, mitophagy

## Abstract

Zearalenone (ZEA) is a common estrogenic mycotoxin in rabbit breeding that causes various toxic effects. Selenomethionine (SeMet) is a feed additive with potent anti-inflammatory and antioxidant properties. To evaluate the protective role and action mechanism of SeMet against ZEA-induced liver injury, 90-day-old rabbits were randomized into five groups: control, ZEA-alone, and SeMet pretreatment at 0.2, 0.35, and 0.5 mg/kg. SeMet was administered for 21 days, followed by continuous intragastric ZEA (1.2 mg/kg B.W.) for 7 days starting on day 15. As a result, ZEA exposure significantly elevated liver function parameters, disrupted lobular architecture, and impaired glycogen synthesis. It also induced liver oxidative stress, thus upregulating expressions of Bax, Cyt C, Caspase-3, and Caspase-9, triggering hepatocyte apoptosis, mitochondrial damage, and mitophagy. SeMet pretreatment activated SIRT1, reduced the acetylated FOXO1/P53 levels, and enhanced CAT and SOD2 expression, mitigating ZEA-induced oxidative stress, apoptosis, and mitophagy. Based on the above findings, SeMet’s alleviating effect might be mediated via the SIRT1-FOXO1/P53 pathway, with 0.35 mg/kg of SeMet exerting the optimal efficacy, highlighting its therapeutic potential for mitigating ZEA-induced hepatotoxicity in rabbits.

## 1. Introduction

Mycotoxins, toxic metabolites produced by molds, can be transferred and bio-accumulated through the food chain, threatening human and animal health and feed safety [1]. Raw feed materials like corn and cereals are highly susceptible to mycotoxin contamination throughout the supply chain. Mycotoxin contamination, such as the common zearalenone (ZEA or F-2 toxin), significantly reduces feed quality by undermining its nutritional value and palatability, which thus adversely influences animal growth performance. In rabbit breeding, exposure to ZEA in feed has toxic effects on the functions of the liver, kidneys, and reproductive system [2]. The liver is both the primary site of ZEA metabolism and a key target organ for it. ZEA can cause swelling and granular degeneration of hepatocytes in weaned piglets [3], and can also lead to diffuse necrosis in mouse liver [4]. This hepatotoxic effect may be closely related to the imbalance of redox homeostasis caused by ZEA. Research indicates that ZEA primarily induces liver injury in mice by activating the ferroptosis pathway, which elevates liver injury markers (ALT/AST) and exacerbates oxidative stress [5]. Additionally, ZEA upregulates pro-apoptotic proteins Bax and Bid via the Fas/FasL pathway, thus inducing cell apoptosis [6]. Despite this, research on how to mitigate ZEA-induced liver injury remains limited.

Given the central role of oxidative stress in liver injury, enhancing hepatic antioxidant capacity emerges as a pivotal intervention strategy. Selenium (Se), an essential trace element, is vital for antioxidant defense and detoxification, acting as the active center of enzymes like GSH-Px and thioredoxin reductase (TrxR), which protect cells from the damage induced by oxygen free radicals [7]. As the important selenium reservoir, the liver concentrates more selenium than other organs. Studies have revealed that Se deficiency causes hepatocyte necroptosis, leading to hepatitis and cirrhosis, which underscores its key role in maintaining liver homeostasis [7]. Selenium exists primarily as inorganic (selenite, selenate) and organic (selenomethionine) forms [8]. Compared with inorganic selenium, selenomethionine (SeMet) offers greater safety, superior bioavailability, and diverse biological functions, making it the preferred selenium source in animal feed. Evidence demonstrates that SeMet protects against diverse hepatotoxic insults, including fluoride- and H_2_O_2_-induced damage, as well as DON-induced toxicity via the ferroptosis and JNK MAPK pathways [9,10,11,12]. Although the protective effect of SeMet against ZEA-induced hepatotoxicity has been preliminarily demonstrated [13], its underlying molecular mechanisms, particularly oxidative stress and apoptosis, remain to be further elucidated. Given the central role of the SIRT1-FOXO1/P53 pathway in redox balance and apoptotic regulation during xenobiotic-induced liver injury, it represents a critical, uninvestigated axis for deciphering the hepatoprotective mechanism of SeMet.

Sirtuin 1 (SIRT1), a crucial deacetylase, modulates cellular homeostasis by targeting key transcription factors like P53 and FOXO1. Its functional characteristic suppressing pro-apoptotic signaling while enhancing antioxidant defenses closely aligns with the biological effect of SeMet [14]. Specifically, SIRT1-mediated deacetylation of FOXO1 enhances its DNA-binding ability, thus regulating the antioxidant enzymes to alleviate oxidative stress [15]. In parallel, deacetylation of P53 by SIRT1 represses its transcriptional activity, thereby protecting cells from oxidative damage and inhibiting apoptosis [16]. Importantly, SIRT1 activation has been shown to alleviate acute alcoholic liver injury by suppressing oxidative stress and apoptosis, highlighting its potential as a therapeutic target in liver injury [17].

Based on the above analysis, SeMet may alleviate ZEA-induced liver injury in rabbits via SIRT1 signaling. Consequently, this study aims to explore the hepatoprotective mechanism of SeMet through SIRT1 regulation, thus providing a scientific basis for its use as a functional feed additive and novel insights into mitigating ZEA-induced hepatotoxicity.

## 2. Materials and Methods

### 2.1. Chemicals and Reagents

In this study, ZEA was purchased from Tianjin Alta tech Co., Ltd. (Tianjin, China); SeMet was sourced from Sangon (Shanghai, China); olive oil was obtained from Olive (Roma, Italy). Paraformaldehyde (4%), glutaraldehyde (2.5%), and sodium pentobarbital (3%) were procured from Dulaimi Biotechnology Co., Ltd. (Wuhan, China). Commercial assay kits, including the BCA Protein Assay Kit (G2026-1000T), T-SOD (G4306-96T), and MDA (G4300-96T) were supplied by Servicebio technology Co., Ltd. (Wuhan, China). Kits for GSH-Px (S0058) and T-AOC (S0116) were from Beyotime (Shanghai, China). TRIzol Reagent was provided by CWBIO Technologies (Beijing, China). The cDNA Synthesis Kit (G3330-100) and SYBR Premix Ex Taq™ Kit (G3326-15) were also from Servicebio. All primary antibodies were sourced from Servicebio, and secondary antibodies were procured from Proteintech (Wuhan, China). Other chemicals and agents were of the highest available purity.

### 2.2. Animals and Experimental Design

**Figure 1:**
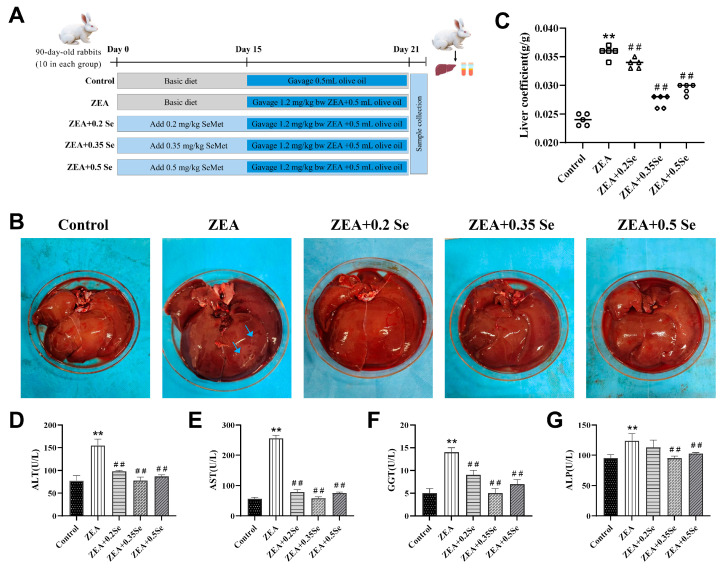
Liver morphology and function index of rabbits (n = 5). (**A**) Grouping of experimental animals. (**B**) Liver morphology (blue arrows: necrotic area). (**C**) Hepatic index. (**D**–**G**) Serum ALT, AST, GGT, and ALP activities. Data are expressed as mean ± SD. ** *p* < 0.01 vs. control group; ## *p* < 0.01 vs. ZEA group.

Fifty 90-day-old healthy Ira rabbits (3.5 ± 0.1 kg) were obtained from the Animal Experiment Center of the Henan University of Science and Technology. After 7 days of acclimatization (21~25 °C, 50~70% relative humidity), they were randomly assigned to five groups (ten per group, with an equal number of male and female rabbits): Control, ZEA (1.2 mg/kg B.W.) and three groups receiving the basal diet supplemented with low- (0.2 mg/kg), medium- (0.35 mg/kg), or high-dose (0.5 mg/kg) SeMet. During the entire period, all rabbits were fed 200 g/day of a standard diet (Table S1) with water and libitum. From days 1–21, three SeMet groups received their respective supplemented diet [18], while the other two groups were fed the basal diet. Starting on day 15, rabbits in the ZEA group and all three SeMet groups were orally gavaged daily for 7 days with 0.5 mL of olive oil containing 1.2 mg/kg of BW ZEA; the control group received an equal volume of olive oil alone (Figure 1A). All procedures were approved by the Henan University of Science and Technology Experimental Animal Ethics Committee (approval No. 4103110414723). No animals in this study experienced any unwarranted suffering.

### 2.3. Sample Collection

At the end of the trial, rabbits were fasted for 12 h and anesthetized by intravenous injection with 3% sodium pentobarbital. Blood was collected via cardiac puncture and then centrifuged at 3000× *g* for 15 min. The resulting serum was then stored at −20 °C for subsequent analysis. Livers were promptly exercised and weighed for the calculation of liver index using the following formula: Liver index (g/kg) = liver weight (g)/body weight (kg) [19]. Sections of liver tissue were fixed in either 4% paraformaldehyde for histological analysis or 2.5% glutaraldehyde (pH 7.4) for transmission electron microscopy. The remaining tissues were stored at −80 °C for other assays.

### 2.4. Serum Biochemical Analysis

Serum biochemical parameters, such as aspartate aminotransferase (AST), alanine aminotransferase (ALT), alkaline phosphatase (ALP), and gamma-glutamyl transferase (GGT), were measured using an automatic biochemical analyzer (BS-240Vet, Mindray, China) with commercial kits [20].

### 2.5. Liver Antioxidant Capacity Testing

Liver tissue samples were homogenized in ice-cold PBS buffer, followed by centrifugation at 12,000× *g* for 15 min at 4 °C. The supernatant was carefully separated and utilized for quantification of total antioxidant capacity (T-AOC), total superoxide dismutase (T-SOD) and glutathione peroxidase (GSH-Px) activities, and methane dicarboxylic aldehyde (MDA) level using the assay kits. All procedures were strictly performed in accordance with the manufacturers’ instructions.

### 2.6. Histological Observation

Liver specimens were immobilized in 4% paraformaldehyde for 24 h, washed, and processed through graded ethanol series and xylene before paraffin embedding. Sections (5 μm-thick) were stained with H&E, Masson, and PAS following standard protocols and observed under a light microscope (ECLIPSE-C1, Nikon, Tokyo, Japan). For pathological evaluation, five fields per section were scored blindly (0–2) for features: unclear contours of hepatic cords, hepatic sinusoidal congestion, fatty degeneration, and lobular inflammation. The scoring was as follows: 0 for none, 1 for mild, and 2 for severe, with a maximum possible total score of 8 [21]. Masson- and PAS-stained areas were quantified using ImageJ software (version 1.54) on five random fields per sample, which were used for calculating the proportion of their total areas occupied.

### 2.7. Ultrastructural Observation

Fresh liver tissues (1 mm^3^) were fixed with 2.5% glutaraldehyde (pH 7.4), dehydrated in a graded ethanol series, osmotically embedded in epoxy resin, and sectioned into ultrathin slices. The sections were then stained with uranyl acetate and lead citrate, then observed under a transmission electron microscope (HT7700, HITACHI). High-resolution images were captured for analysis [22].

### 2.8. Quantitative Real-Time PCR

RNA extraction and RT-qPCR were performed following the protocols established in our lab [23]. Briefly, total RNA was extracted from frozen liver tissues using Trizol reagent and reverse-transcribed into cDNA. Quantitative PCR was carried out using SYBR^®^ Premix Ex Taq™ on a thermal cycler. Relative mRNA expression of target genes was calculated using the 2^−∆∆Ct^ method, with β-actin as the endogenous control. (Note: β-actin was used as an endogenous control based on similar model experiments [12]). Primer sequences are listed in Table S2.

### 2.9. Immunohistochemistry

Immunohistochemical (IHC) staining for SIRT1, FOXO1, CAT, and SOD2 was performed on paraffin-embedded sections. After deparaffinization and rehydration, endogenous peroxidase was quenched with 3% H_2_O_2_ at room temperature. Antigen retrieval was conducted in 0.01 M of citrate buffer (pH 6.0), followed by blocking with 3% BSA at 37 °C for 30 min. Sections were incubated with primary antibodies at 4 °C overnight and then with a biotinylated secondary antibody IgG at 37 °C for 50 min. Signal was developed using the freshly prepared DAB, counterstained with hematoxylin and examined under a light microscope. Protein expressions were quantified as the average integrated optical density (IOD) of the positive cells.

### 2.10. Immunofluorescence

Paraffin-embedded sections were dewaxed and rehydrated and subjected to antigen retrieval in EDTA buffer (pH 8.0) using microwave heating. Following blocking with 3% BSA at room temperature for 30 min, sections were incubated overnight at 4 °C with primary antibodies against Parkin and P62, followed by a Cy3-conjugated secondary antibody for 50 min in the dark. Nuclei were stained with DAPI for 10 min in the dark, followed by autofluorescence quenching for 5 min. Sections were mounted for imaging under a fluorescence microscope (Eclipse C1, Nikon) [24].

### 2.11. Western Blotting Assay

Western blot was performed to analyze the expressions of proteins related to antioxidant signaling (SIRT1, FOXO1 etc.), apoptosis (Caspase-3, -9 etc.), and mitophagy (Pink1, P62 etc.) in rabbit liver tissues, with β-actin as the internal control. Primary antibody dilutions are listed in Supplementary Table S3. Specifically, liver tissues (30 mg/sample) were homogenized in RIPA lysis buffer containing 1% (*v*/*v*) 100 mM PMSF, lysed on ice for 30 min, and centrifuged at 3000× *g* for 10 min at 4 °C. Protein concentration was quantified by BCA assay. Samples (40 μg) were separated by 10% SDS-PAGE and electro transferred onto a PVDF membrane (Millipore, Billerica, MA, USA). After blocking with 5% (*w*/*v*) non-fat milk, the membrane was incubated with primary antibodies overnight at 4 °C, followed by the HRP-conjugated secondary antibody IgG (1:3000). Protein bands were visualized using ECL reagent, and grayscale values were analyzed in ImageJ software and normalized to β-actin [18].

### 2.12. Statistical Analysis

This study employed a completely randomized design with each rabbit serving as an independent experimental unit (n = 5 per group). Data were analyzed using one-way ANOVA with IBM SPSS Statistics 25.0 (IBM Corp., Chicago, IL, USA) to evaluate treatment effects, followed by Tukey’s HSD test for multiple comparisons. Figures were generated using GraphPad Prism 8 (GraphPad Software, San Diego, CA, USA). Results are presented as mean ± standard deviation (SD). Statistical significance between groups was defined as */# *p* < 0.05 (significant difference) and **/## *p* < 0.01 (extremely significant difference).

## 3. Results

### 3.1. SeMet Alleviates ZEA-Induced Liver Function Damage in Rabbits

To preliminarily assess the impact of SeMet on liver tissues of rabbits treated with ZEA, liver morphology and function were assessed. The results showed (Figure 1B–G) that, compared with the control, the ZEA-exposed group exhibited enlarged liver volume, surface necrotic areas, and a significantly increased liver coefficient (*p* < 0.01). Concurrently, serum ALT, AST, GGT, and ALP activities exhibited a significant increase (*p* < 0.01). Meanwhile, SeMet pretreatment not only effectively restored liver appearance and coefficient, but also reduced enzyme activities to varying degrees (*p* < 0.01). Specifically, low-, medium-, and high-dose SeMet reduced ALT by 36.8%, 49.7%, and 44.1%; AST by 69.2%, 77.5%, and 70.6%; GGT by 35.7%, 64.3%, and 50.0%; and ALP by 8.9%, 23.1%, and 20.8%, respectively (*p* < 0.01). These results demonstrate that SeMet can alleviate the abnormal conditions of the liver in rabbits challenged to ZEA.

### 3.2. Effect of SeMet on Liver Morphology and Structure in Rabbits Exposed to ZEA

**Figure 2:**
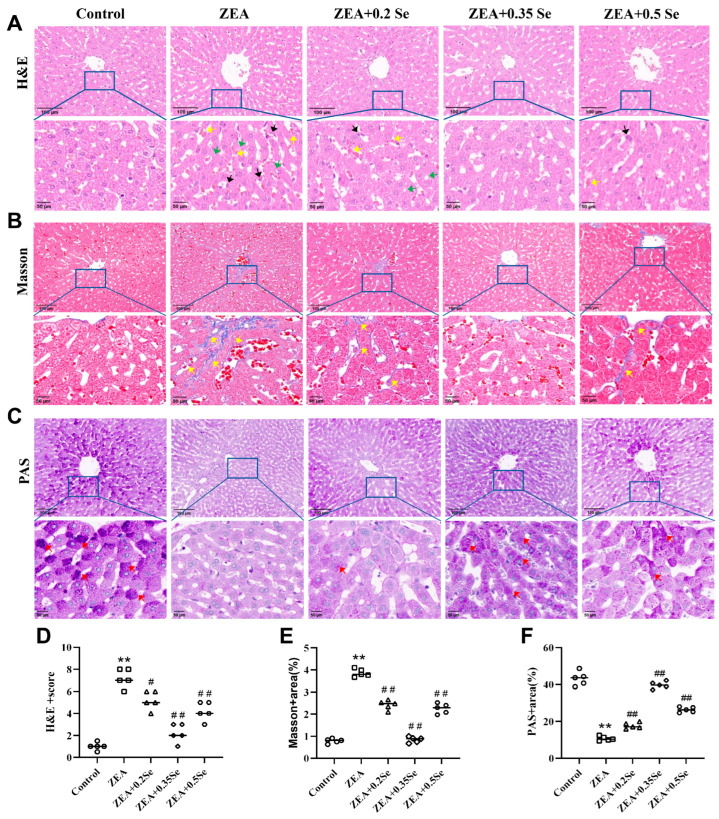
Histological changes in rabbit liver tissue (n = 5). (**A**) H&E staining showing inflammatory cell infiltration (black arrow), steatosis (green arrow), and sinusoidal congestion (yellow arrow). (**B**) Masson staining indicating collagen fiber deposition (yellow arrow). (**C**) PAS staining revealing purple–red glycogen granules (red arrow). The top row shows images at ×100 magnification (scale bar = 100 μm); the bottom row displays corresponding enlarged views at ×200 (scale bar = 50 μm). (**D**) HE stains score. (**E**,**F**) Positively stained areas in Masson and PAS staining. Data are expressed as mean ± SD. ** *p* < 0.01 vs. control group; # *p* < 0.05, ## *p* < 0.01 vs. ZEA group.

To further elucidate the adverse effects of ZEA on rabbit liver and the protective role of SeMet, histological analysis (H&E, Masson, and PAS staining) was performed to investigate the structural alterations in liver tissue. As a result, the control group showed no significant pathological changes. In contrast, the ZEA group exhibited disrupted liver lobule structure, unclear hepatocyte cord contours, dilated and congested sinusoids, inflammatory infiltration, steatosis, and a significant reduction in purple–red glycogen granules (Figure 2A,C). Furthermore, significant deposition of collagen fibers was observed in both the portal and central venous areas (*p* < 0.01, Figure 2B,E), indicating a marked elevation in liver fibrosis levels, which was confirmed by the analysis of collagen volume fraction (*p* < 0.01, Figure 2C,F). However, SeMet pretreatment improved these pathological changes to varying degrees, with the medium dose most significantly restoring liver glycogen content (*p* < 0.01, Figure 2C,F). Nonetheless, mild to moderate alterations, such as sinusoid dilation and minimal collagen deposition in the central venous region, were present in the low- and high-dose SeMet groups.

### 3.3. Effect of SeMet on Oxidative Stress in Rabbits’ Liver Exposed to ZEA

**Figure 3:**
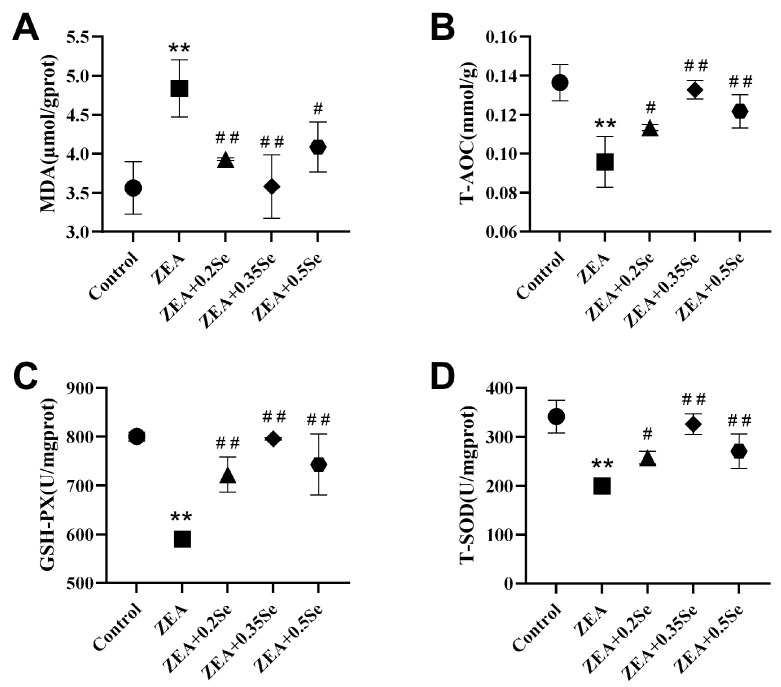
Oxidative stress parameters in rabbit liver (n = 5). (**A**) MDA content. (**B**) T-AOC level. (**C**) GSH-Px activity. (**D**) T-SOD activity. Data are expressed as mean ± SD. ** *p* < 0.01 vs. control group; # *p* < 0.05, ## *p* < 0.01 vs. ZEA group.

To assess hepatic redox status, this study measured the levels of MDA, T-AOC, and the activities of antioxidant enzymes GSH-Px and SOD. As shown in Figure 3, ZEA exposure significantly increased oxidative stress in rabbit livers, characterized by a remarkable increase in MDA content *(p* < 0.01) and a substantial decrease in T-AOC, GSH-Px, and SOD activities (*p* < 0.01) compared to the control. However, all three doses of SeMet significantly attenuated this effect, reducing MDA by 18.7%, 26.0%, and 15.5%, respectively *(p* < 0.05), while upregulating T-AOC, GSH-Px, and T-SOD activities (*p* < 0.05). These results indicate that SeMet supplementation can effectively improve ZEA-induced oxidative stress in rabbit liver.

### 3.4. Impact of SeMet on SIRT1-FOXO1 Pathway in Rabbit Liver Exposed to ZEA

**Figure 4:**
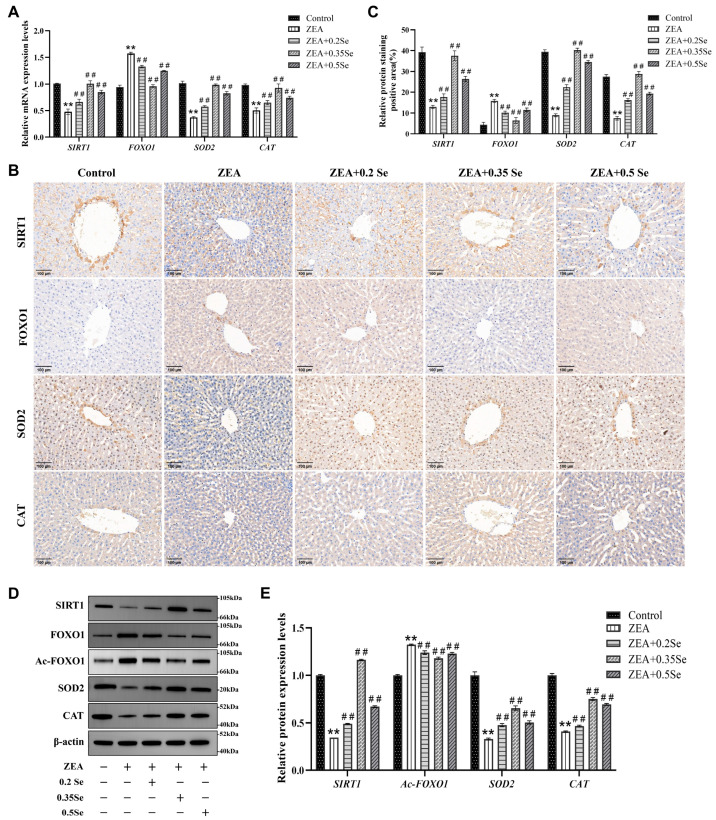
Expression of SIRT1-FOXO1 pathway components in rabbit liver tissue (n = 5). (**A**) mRNA levels of SIRT1, FOXO1, SOD2, and CAT determined by RT-qPCR. (**B**) Representative immunohistochemical image (×100 magnification; scale bar = 100 μm). (**C**) Semiquantitative analysis of immunohistochemical analysis. (**D**) Western blot bands of proteins. (**E**) Quantitative analysis of protein expression. Data are expressed as mean ± SD. ** *p* < 0.01 vs. control group; ## *p* < 0.01 vs. ZEA group.

To further investigate the protective mechanism of SeMet against ZEA-induced hepatic oxidative damage, this study analyzed the SIRT1-FOXO1 pathway using RT-qPCR, immunohistochemistry and Western blot. The results, as illustrated in Figure 4, revealed that compared to the control, mRNA levels of SIRT1 and antioxidant enzyme genes CAT and SOD2 were dramatically downregulated in the ZEA group (*p* < 0.01). In contrast, FOXO1, a key target gene of SIRT1, was markedly upregulated (*p* < 0.01). In three SeMet- pretreated groups, SIRT1, CAT, and SOD2 transcript levels were notably restored (*p* < 0.01), while FOXO1 expression was suppressed (Figure 4A). Consistent with transcriptional changes, protein levels of SIRT1, FOXO1, CAT, and SOD2 showed similar trends (Figure 4B–E). Additionally, this study evaluated the acetylation level of the FOXO1 protein, revealing that the relative level of Ac-FOXO1 protein was significantly reduced by 6.3–10.8% compared to the ZEA group (*p* < 0.01, Figure 4E). These results suggest that SeMet ameliorates ZEA-induced oxidative damage by modulating the SIRT1-FOXO1 pathway in rabbit livers.

### 3.5. Effect of SeMet on Apoptosis-Related Gene and Protein Expression in ZEA-Exposed Rabbit Liver

**Figure 5:**
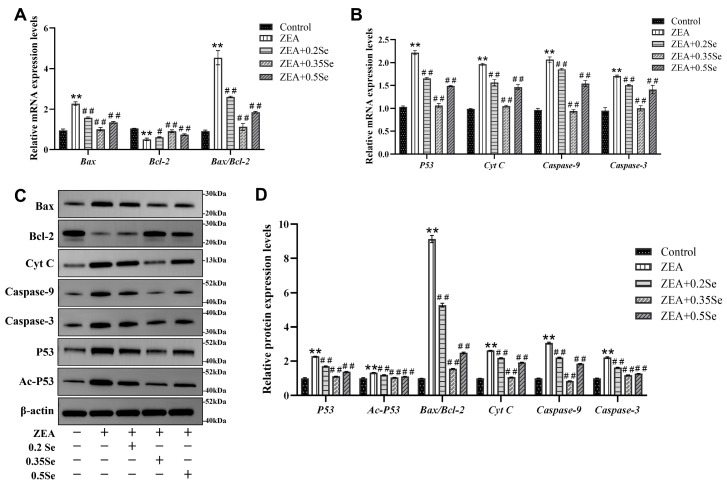
Expression of apoptosis-related genes and proteins in rabbit liver cells (n = 5). (**A**,**B**) mRNA levels of Bax, Bcl-2, Cyt C, Caspase-9, Caspase-3, and P53 by RT-qPCR analysis. (**C**) Western blotting band of proteins. (**D**) Quantitative analysis of protein expression, including the Bax/Bcl-2 ratio. Data are expressed as mean ± SD. ** *p* < 0.01 vs. control group; # *p* < 0.05, ## *p* < 0.01 vs. ZEA group.

Because oxidative stress may further induce the occurrence of cell apoptosis, the present study examined apoptosis-related gene and protein expression. The results showed that mRNA levels of pro-apoptotic genes (Bax, Cyt C, Caspase-9, Caspase-3, and P53) were significantly elevated, and anti-apoptotic Bcl-2 mRNA level was markedly reduced in the ZEA-treated group compared to the control (*p* < 0.01). However, SeMet pretreatment reversed these changes, with significant downregulation of pro-apoptotic transcripts and upregulation of Bcl-2 (*p* < 0.05; Figure 5A,B). Corresponding protein expressions showed similar patterns (Figure 5C,D). Compared to the control, the ZEA-treated group exhibited a significant elevation of pro-apoptotic proteins (Bax, Cyt C, Caspase-9, Caspase-3, Ac-P53, and P53) and the strong downregulation of Bcl-2 (*p* < 0.01). In SeMet-pretreated groups, pro-apoptotic protein levels were significantly reduced (*p* < 0.01). Due to the close correlation between the Bax/Bcl-2 ratio and Ac-P53 in cell apoptosis, these parameters were quantitatively analyzed. Results revealed that, compared to the ZEA group, the Bax/Bcl-2 ratio decreased significantly by 42.4%, 83.0%, and 72.7% in low-, medium-, and high-dose groups, respectively (*p* < 0.01). Ac-P53 protein levels were also reduced by 10.6%, 21.3%, and 16.8% (*p* < 0.01). These data suggest that SeMet attenuates ZEA-induced hepatocyte apoptosis by modulating the mitochondrial pathway.

### 3.6. Effect of SeMet on Hepatocyte Mitochondrial Structure in ZEA-Exposed Rabbit Liver

**Figure 6:**
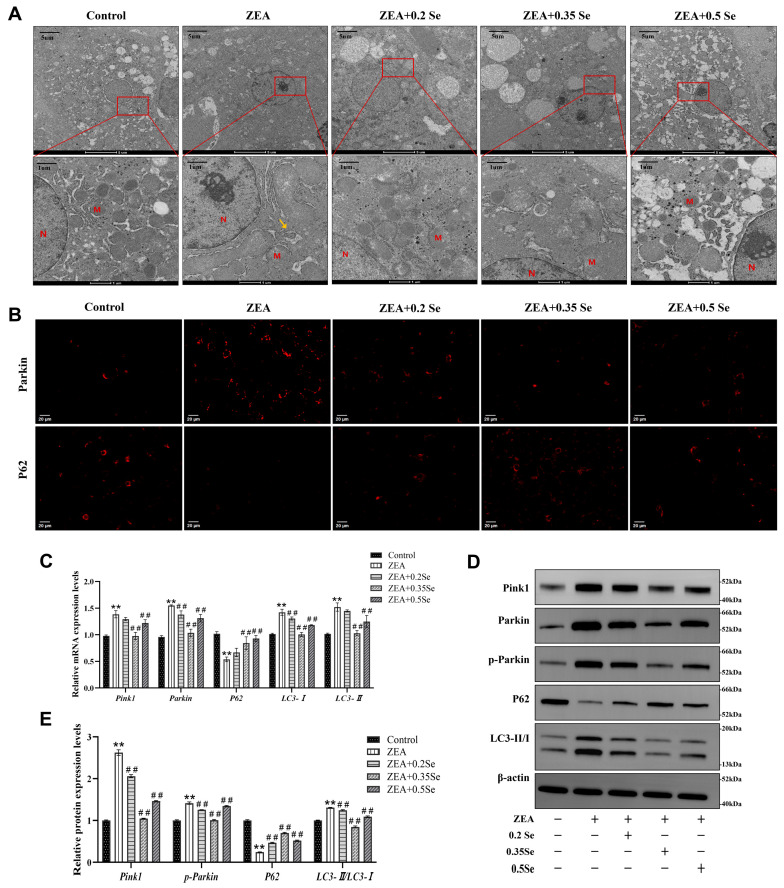
Changes in mitochondrial ultrastructure and mitophagy markers in rabbit hepatocytes (n = 5). (**A**) TEM images of mitochondrial ultrastructure. M: Mitochondria; N: Nucleus; Yellow arrow: Autophagosome. (**B**) Immunofluorescence localization of Parkin and P62 (Cy3-labeled). (**C**) mRNA expression levels of Pink1, Parkin, P62, LC3-I, and LC3-II measured by RT-qPCR. (**D**) Western blot bands of Pink1, Parkin, p-Parkin, P62, and LC3-II/LC3-I. (**E**) Quantitative analysis of mitophagy-related protein expression. Data are expressed as mean ± SD. ** *p* < 0.01 vs. control group; ## *p* < 0.01 vs. ZEA group.

Oxidative stress and cell apoptosis are usually associated with mitochondrial structural damage. For this reason, the ultrastructure of hepatocyte mitochondria was examined using transmission electron microscopy (TEM). As illustrated in Figure 6A, the hepatocyte mitochondria in the control group were structurally intact and evenly distributed in the cytoplasm. In contrast, ZEA exposure led to severe impairment in mitochondrial ultrastructure, as evidenced by swelling, cristae disruption, and the presence of autophagosomes (yellow arrows). Supplementation with SeMet partially mitigated these alterations, increasing mitochondrial number and restoring morphological integrity.

### 3.7. Effect of SeMet on Expression of Mitophagy Factors in ZEA-Exposed Rabbits’ Liver

Mitochondrial damage can trigger the activation of autophagy, the cellular process that captures and degrades the impaired mitochondria, attenuating cell apoptosis. Thus, this study assayed key autophagy markers via RT-qPCR, immunofluorescence, and Western blot. RT-qPCR results revealed that, compared to the control group, ZEA exposure caused significantly increased mRNA levels of Pink1, Parkin, LC3-I, and LC3-II while they decreased P62 level (*p* < 0.01, Figure 6C). SeMet pretreatment, particularly at medium and high doses, significantly reversed these changes and led to the downregulation of Pink1, Parkin, and LC3-II, as well as a significant upregulation of P62 (*p* < 0.01).

Parkin and P62 are pivotal proteins in mitophagy activation. Immunofluorescence analysis showed that ZEA exposure caused a significantly increased Parkin while P62 fluorescence intensity decreased. SeMet pretreatment reversed these alterations, most effectively in the medium-dose SeMet group (Figure 6B). Furthermore, Western blot analysis confirmed ZEA-induced mitophagy, as evidenced by the significant elevated autophagy markers LC3-II/LC3-I, Pink1, and p-Parkin, and the reduced P62 protein level (*p* < 0.01; Figure 6D,E). In contrast, SeMet pretreatment partially restored the expression of these mitophagy markers.

## 4. Discussion

ZEA, a widespread estrogenic mycotoxin, poses serious threats to rabbit health, inducing reproductive, immune, and hepatic toxicities through dietary exposure. As the important site of ZEA metabolism, the liver is particularly susceptible to this toxin. Consistent with previous reports [20], ZEA challenge in our study increased liver coefficient; enhanced serum ALT, AST, GGT, and ALP activities; and provoked histopathological damage including disordered structure of liver lobule, hepatic sinusoid congestion, fibrosis, and glycogen depletion. Collectively, these observations underscore the hepatotoxic effects of ZEA in rabbits. Despite extensive research on mitigating ZEA’s liver toxicity, there remains an urgent need for new and effective treatments. SeMet, an organic selenium compound recognized for its strong antioxidant activity and high bioavailability, demonstrated effective hepatoprotection in our study. Dietary SeMet supplementation significantly ameliorated ZEA-induced liver injury, as reflected by recovery of hepatic coefficients, serum biomarkers, and tissue structure. Notably, the medium-dose SeMet exhibited the most pronounced protective efficacy, suggesting its potential as an optimal intervention, which aligned with findings by previous reports [25].

Oxidative stress is recognized as a contributor to xenobiotic-induced liver injury. Under physiological conditions, redox homeostasis is maintained between ROS production and the cellular antioxidant system. However, ZEA has been shown to break this equilibrium, leading to oxidative stress, as reported in mice [26]. Consistent with this, this study revealed that ZEA significantly elevated hepatic MDA level, while reducing T-AOC and antioxidant enzymes activities (GSH-Px, T-SOD), indicating the impairment of the ROS defense system. Selenium, an essential component of antioxidant enzymes such as GSH-Px and TrxR, plays a central role in redox regulation. In line with a previous study [27], SeMet supplementation effectively attenuated ZEA-induced oxidative stress by lowering MDA level and increasing T-AOC, T-SOD, and GSH-Px activities. Particularly, the medium-dose SeMet exhibited the most pronounced effect, underscoring SeMet’s efficacy in restoring hepatic antioxidant potential. To further explore its protective mechanism, our study examined the SIRT1-FOXO1 pathway, a pivotal regulator of antioxidant defense. SIRT1 deacetylates FOXO1, thus facilitating its binding to and transactivation of downstream targets like SOD2 and CAT. In our experiment, ZEA downregulated the expressions of SIRT1, CAT, and SOD2 in rabbit liver, while upregulating FOXO1 expression. SeMet reversed these alterations, restoring SIRT1 expression, promoting FOXO1 deacetylation, and enhancing antioxidant enzyme levels, which implied that SeMet alleviates ZEA-induced oxidative liver injury via the SIRT1-FOXO1 pathway, corroborating earlier findings that SIRT1 activation protected against H_2_O_2_-induced stress via FOXO1 deacetylation [28].

Studies have established a close link between ZEA-induced oxidative stress and apoptosis. For instance, ZEA treatments (0–100 μM) significantly increased ROS production and apoptosis rates in bovine mammary epithelial cells [29]. Mitochondria, being highly vulnerable to ROS, play a central role in this process. Excessive ROS can trigger a mitochondrial-caspase-dependent apoptotic cascade [30]. Under oxidative stress, ROS accumulation promotes mitochondrial translocation of P53, which upregulates Bax and suppresses Bcl-2, leading to loss of mitochondrial membrane potential (MMP), the release of Cyt C into the cytoplasm, and subsequent activation of Caspase-9 and Caspase-3. In this study, ZEA exposure dramatically increased the expressions of pro-apoptotic factors (P53, Bax, Cyt C, Caspase-9, and Caspase-3) and decreased anti-apoptotic Bcl-2 in rabbit liver, confirming mitochondrial pathway-mediated apoptosis. SeMet supplementation obviously reversed these changes, restoring expression levels of these apoptotic regulators. Furthermore, we found that SeMet-activated SIRT1 not only mitigated oxidative injury by FOXO1 deacetylation but also inhibited mitochondrial apoptosis by deacetylating P53 (Ac-P53). This aligns with previous reports highlighting the SIRT1-P53 axis in liver pathology. For example, dihydroquercetin improved acute liver failure by targeting ferroptosis and mitochondria apoptosis via SIRT1-P53 [31], and vitexin alleviated ethanol-induced liver injury via the same pathway [32]. Our results collectively revealed that SeMet alleviated ZEA-induced apoptotic liver injury via the SIRT1-P53 pathway.

As previously discussed, mitochondria function as both a primary endogenous source and a key target of ROS. Excessive ROS elevates mitochondrial membrane permeability, thus inducing mitochondrial depolarization. Consistent with this mechanism, our study demonstrated that ZEA-induced ROS overproduction triggered this cascade, ultimately causing structural damage. This fostered a detrimental cycle of oxidative stress and mitochondrial injury, culminating in cell apoptosis. Our results further showed that ZEA-triggered oxidative stress also activated mitophagy in rabbit hepatocytes. Substantial efforts have been devoted to identifying strategies that alleviate ZEA-induced damage. For example, the antioxidant resveratrol has been proven to minimize DNA damage and modulate antioxidant enzymes in ZEA-exposed rats. Similarly, resveratrol relieved mitochondrial damage and prevented apoptosis in ZEA-treated oocytes via the Pink1-Parkin-mediated mitophagy pathway [33].

It is well-known that the Pink1-Parkin signaling axis plays a central role in regulating mitophagy. Upon mitochondrial damage, Pink1 accumulates on the outer membrane, recruiting and activating Parkin. Parkin, in turn, promotes the ubiquitination of mitochondrial substrates and facilitates the recruitment of the adaptor P62, which interacts with LC3 to form autophagosomal membranes. The conversion of LC3-I to LC3-II serves as a well-established marker of autophagosome formation [34]. Thus, the levels of LC3-II/LC3-I and P62 proteins are widely used as reliable indicators of autophagic activity. In the ZEA group, we observed mitochondrial swelling and the formation of autophagosome, which provides evidence for the presence of mitophagy. Moreover, this study found that Parkin and P62 are mainly expressed in the cytoplasm, being consistent with mitochondrial localization, which indicates that ZEA-induced damage may activate Pink1-Parkin-mediated mitophagy. This pathway promotes lysosome–autophagosome fusion and degradation of the damaged mitochondria, thus helping maintain cellular homeostasis [35]. Previous studies have confirmed the importance of Pink1-Parkin signaling in mitophagy induced by hepatotoxins, including cadmium, aflatoxin B1, and NaAsO2 [35,36,37].

Intriguingly, our study revealed that the hepatoprotective effect of SeMet was not linearly dose dependent. The medium dose (0.35 mg/kg) provided the strongest protection, while both lower (0.2 mg/kg) and higher (0.5 mg/kg) doses were less effective. This may be attributed to the biphasic nature of Se activity. The low dose was likely insufficient to exert full protective effects, whereas the high dose may interfere with normal selenoprotein synthesis. The blocked synthesis of selenoprotein can compromise antioxidant defense and immune regulation, ultimately exacerbating ROS generation and weakening the protective effect of SeMet [38]. These interpretations, however, remain speculative based on an integrated analysis of our data and the existing literature, and require further validation through studies on SeMet absorption kinetics, metabolic dynamics, and toxicity profiles.

## 5. Conclusions

This study demonstrates that ZEA induces oxidative stress, apoptosis, and mitochondrial damage in rabbit livers, accompanied by the activation of the Pink1-Parkin mitophagy pathway. Dietary SeMet supplementation activates hepatic SIRT1, which in turn attenuates ZEA-induced liver injury via deacetylation of downstream targets FOXO1 and P53. The most effective protection was achieved with a medium SeMet dose (0.35 mg/kg). These findings highlight SIRT1 as a potential therapeutic target, offering novel mechanistic insights into SeMet-mediated protection against ZEA-induced liver injury.

This study initially used β-actin as a single reference gene, which was subsequently validated. Future experiments will pre-screen a reference gene panel.

## Data Availability

The original contributions presented in this study are included in the article/Supplementary Material. Further inquiries can be directed to the corresponding author.

## Supplementary Materials

The following supporting information can be downloaded at: https://www.mdpi.com/article/10.3390/antiox15020176/s1, Table S1: Ingredients and chemical composition of basal diets. Table S2: Primer Sequences for the interested genes. Table S3: Primary antibody information.
